# Thermosets from Birch Bark: A Holistic Approach Using Green Solvents and Processes

**DOI:** 10.1021/acsomega.5c05162

**Published:** 2025-07-28

**Authors:** Megan L. Dodge, Luke Goodhope, Qwin Pisacane, Rongmin Tang, Sophia I. Harrill, Heather M. LaFrance, Alexandra M. Lehman-Chong, Joseph F. Stanzione, Lindsay Soh, Melissa B. Gordon

**Affiliations:** † Department of Chemical and Biomolecular Engineering, 3091 Lafayette College, 740 High Street, Easton, Pennsylvania 18042, United States; ‡ Integrative Engineering Program, Lafayette College, 740 High Street, Easton, Pennsylvania 18042, United States; § Department of Chemical Engineering, 3536 Rowan University, 201 Mullica Hill Road, Glassboro, New Jersey 08028, United States; ∥ Advanced Materials & Manufacturing Institute (AMMI), Rowan University, 201 Mullica Hill Road, Glassboro, New Jersey 08028, United States

## Abstract

Many recent efforts toward sustainable polymer development use building blocks from renewable biomass feedstocks. However, issues arising from the processes used to extract starting materials from biomass are often overlooked, despite the safety and environmental hazards associated with energy-intensive separation processes and solvent utilization. Here, we describe a holistic approach toward using green solvents and processes to synthesize polyester thermosets from birch bark, a waste product from the paper and pulp industry. Betulin, a diol with a pentacyclic ring structure, was extracted from the bark of silver birch trees via reflux boiling using green solvents available from biobased sources. Ethanol and 1:1 ethanol:ethyl acetate mixtures were effective solvents for extraction, with additional selectivity achieved via antisolvent precipitation. Betulin-rich extracts containing 62.2–81.5 wt % betulin were produced and directly used to prepare polyester thermosets using one-pot, solventless polycondensations with 100% of the starting materials available from biomass feedstocks. The polymers prepared directly from extracts had properties comparable to those synthesized from pure betulin, suggesting that additional processing steps required to achieve higher purity betulin may not be warranted. Overall, we present an approach to polyester development from betulin-rich birch bark extracts, which incorporate green chemistry and engineering principles from feedstock to product.

## Graphical abstract

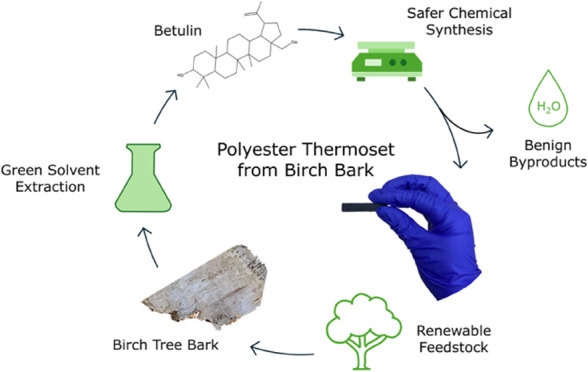

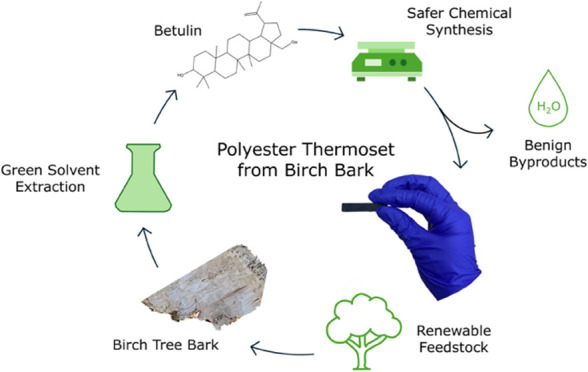

## 1 Introduction

Polyesters are commonly used in plastics, coatings, fibers, and elastomers, yet their production presents pressing environmental challenges due to their reliance on fossil fuel feedstocks and energy-intensive extraction and refining practices.^1^ In order to design greener polyester alternatives, a comprehensive approach that takes into account the principles of green chemistry and engineering^2,3^ will increase the likelihood of avoiding negative impacts associated with polyester sourcing and manufacturing.^4^ Utilizing biomass as a feedstock for the production of biobased polyester alternatives may also carry potential advantages stemming from the inherent biobased monomer structure to achieve polymer properties suitable for a variety of applications. Furthermore, careful choice of processing parameters (e.g., catalyst, solvents, conditions) may decrease hazards and impacts during manufacturing.^5^ These sourcing and production considerations, choice of biomass feedstock, extraction technique, and synthetic approach, are critical for sustainable biobased polyester development.

Birch bark is a promising, renewable feedstock and a valuable source of extractable building blocks for biobased polymer development. The outer layer of the bark is shed naturally from the tree and is a rich source of betulin, a nontoxic aliphatic diol with a pentacyclic ring structure. Betulin can be extracted at quantities up to 30 dry wt % from the outer bark, depending upon the species of birch, season, location, and age.^6–9^ The inherent functionality of this triterpenoid lends itself for use as a monomer in polyester synthesis.^10,11^ Betulin can undergo polycondensation reactions as a diol and may offer rigidity and thermal stability to polymers due to its bulky ring structure. While birch bark is a waste product from paper and pulp production,^12^ recent studies have valorized the use of betulin for polyester development using sustainable and industrially viable approaches.^13,14^ Previously, we synthesized high-performance polyester thermoplastics and thermosets from betulin using a series of biobased linear aliphatic diacids via melt polycondensation, avoiding the use of corrosive comonomers and solvents.^13^ To expand the scope of applications, we explored the use of itaconic acid as a biobased and commercially available comonomer to prepare betulin-based thermosets using two preparation methods. Both UV and thermally curable thermosets were synthesized with tunable properties based on monomer content.^14^ These studies illustrate the promise of betulin-based polyesters; however, these syntheses use pure (≥98%) betulin requiring elaborate refining procedures for separation from other easily extractable molecules including other triterpenoids (e.g., lupeol, betulinic acid) as well as sugars, phenolics, and suberin.^6^

The method for isolating and purifying betulin from birch bark may impact the extraction yield, polymer properties, and environmental implications of the process. Extraction involves the use of solvents (e.g., alcohols to alkanes and halogenated solvents)^6,15–18^ for product separation and fractionation. The solvents are applied via various extraction methods including Soxhlet extraction, reflux boiling, steam distillation, and/or high temperature conditions as well as alkaline conditions^19^ to enable high extraction yields from the complex biomass matrix, preferably with high betulin selectivity.^6,15^ Supercritical CO_2_ with modifiers has also been used to extract betulin from biomass but requires large amounts of solvent with low recoveries.^17,20^ The trade-offs between the different extraction solvents and methods are evident as improvements in the extract yield or purity of betulin typically come at a cost of increased processing waste, energy use, and solvent toxicity.^6,17^ For example, the method of Šiman et al. consists of six process steps and requires several hazardous solvents (e.g., benzene and chloroform) but produces a betulin product of greater than 99% purity.^16^ Greener solvents (e.g., acetone, alcohol, ethyl acetate, and water mixtures)^6^ have been evaluated for their extraction effectiveness; while these organic solvents have decent recoveries, removal of excess solvent, long extraction times, high temperatures, as well as the nonselective extraction of a large number of molecules from biomass warrant the exploration of a more effective, greener approach. Fridén et al. included a precipitation step for betulin recovery from ethanol extracts by using water as an antisolvent and found increases in extract selectivity (60.9% versus 33.9% mass of betulin per mass of dry extract, with and without precipitation, respectively) though the yield did slightly decrease (4.75 wt % compared to 5.16 wt % mass of betulin per mass of birch bark, respectively).^17^ The choice of solvent and processing method will affect extract composition and may impact polymer properties. For example, Guidotti et al. investigated triterpenoid and suberin extracts from birch bark to create poly­(hexamethylene furanoate) blends illustrating that properties are affected by the relative amounts of extract components and can lead to improved properties.^21^ Here, we seek to embrace the embedded complexity of biomass systems and better understand the impact of solvent and extraction methods on polymer synthesis and properties.

In this study, we demonstrate the use of green solvents and processes to produce betulin-rich extracts from birch bark and their direct use for the synthesis of biobased polyester thermosets. The work addresses a gap in the current literature connecting how the extraction process and extract composition affect the properties of biobased polymers. The solvents applied herein (ethanol, ethyl acetate, and water) are available from biobased sources and considered green solvents.^22,23^ Using reflux boiling and antisolvent precipitation, extracts high in betulin content were incorporated into polyester thermosets via melt polycondensation with a biobased diacid comonomer and glycerol as the cross-linker. Our overall process demonstrates an effective route for polyester synthesis from bark extract that considers both functionality and environmental sustainability. Furthermore, we investigated the relationship between the extraction method and solvent choice on the betulin composition of the extract and the thermomechanical and degradative properties of the fully cured polyester thermosets. To the best of our knowledge, this is the first report investigating how the extraction method and solvents used to extract betulin from birch bark influence final polymer properties and compare to polymer samples produced using pure betulin. The work presented here provides a strategy for the incorporation of green chemistry and engineering principles through the use of renewable feedstocks, safer solvents for extraction, efficient separations, and less hazardous polymerizations to produce biobased polymers from feedstock to product.

## 2 Materials and Methods

### 2.1 Materials

Betulin (98.0%) was procured from BOC Sciences, stored at −20 °C, and used to fabricate polymer samples to compare to samples prepared using betulin-rich extracts. Glycerol (≥99.5%) was purchased from Sigma-Aldrich and 1,12-dodecanedioic acid (DDDAc, ≥99.0%) was purchased from TCI America. Dibutyltin dilaurate (DBTL, >95.0%) was purchased from TCI America and stored at 5 °C. Miller-Stephenson PTFE release agent (MS-122) was acquired from Grainger. Liquid nitrogen (LN_2_, 99.998%) was procured from Airgas. All solvents were purchased from Thermo Fisher Scientific and were of >99% purity. Bone-dry CO_2_ with a siphon tube was supplied by Airgas, Inc. All chemicals were used as received and stored at room temperature unless otherwise noted.

### 2.2 Preparation of Betulin Extracts

#### 2.2.1 Bark Collection and Preparation

Bark from silver birch trees (*Betula pendula*) was collected locally. Upon harvesting, the bark was stored in a desiccator for at least 48 h. The bark was then milled using a Waring commercial blender. The milled bark (US size 4 mesh, 4.75 mm) was stored below 6 °C until use.

#### 2.2.2 Solvent Screening (Extraction)

Prepared bark (50 mg) and glass beads (50 mg) were placed in a microcentrifuge tube with 1.5 mL of solvent. Each sample was homogenized (BeadBug) at 1800 rpm for 3 min, which was repeated three times. The samples were heated to 60 °C on a heater block for 1 h. Finally, the samples were cooled, and the remaining liquid was removed and diluted for analysis using HPLC.

#### 2.2.3 Supercritical CO_2_ (scCO_2_) Extraction

All scCO_2_ extractions were carried out using an HPR-Series Reactor (Supercritical Fluid Technologies, Inc.) with an impeller. Prepared birch bark (100 mg) and cosolvent were added to the reactor before heating and pressurization. The continuous extraction effluent was set at a flow rate of 150 sccm and was sparged into chilled ethyl acetate.

#### 2.2.4 Extraction and Recovery

An overview of the birch bark to extract process is provided in Figure S1. Briefly, prepared bark (11 g) and 220 mL of solvent (ethanol or 1:1 (vol) EtOH:EtAc) were added to a 500 mL round-bottomed flask for reflux boiling. The flask was attached to a condenser and boiled under reflux for 10 min. The mixture was then cooled to room temperature and filtered to remove the solid bark particles. For samples without antisolvent precipitation, the solvent was evaporated at laboratory temperature under the fume hood. For samples undergoing antisolvent recovery, DI water was added to the extraction solvent. The samples were incubated for 1 h, and the resulting precipitate was filtered and dried in an oven at 50 °C until fully dry.

### 2.3 Extract Characterization

#### 2.3.1 Quantification of Hydroxyl Groups

Hydroxyl numbers in extract samples were quantified using ASTM 222-17, a standard test method for quantifying hydroxyl groups attached to primary and secondary carbon atoms in aliphatic and alicyclic compounds and phenols. Briefly, the sample (or a blank containing no solution) is acetylated in a solution of acetic anhydride at reflux temperature with constant stirring for 1.5 h. The excess reagent is then hydrolyzed with water, creating acetic acid, which is then titrated with a standard sodium hydroxide solution. The hydroxyl number and hydroxyl-containing compound mass percent are calculated from the difference in the titration of a blank and solutions containing samples as detailed in the Supporting Information (SI).

#### 2.3.2 High-Performance Liquid Chromatography (HPLC)

High-performance liquid chromatography (Waters Alliance e2695) was used to separate and quantify triterpenoids in extract samples. The mobile phase composition was 94% ACN and 6% water (v:v) with 0.1% acetic acid at 1.2 mL/min isocratic flow. The separation was done on an XTerra MS C18 Column (125 Å, 5 μm, 4.6 mm × 150 mm). Triplicate samples were dissolved in ethanol at a 0.5 g/L solid to solvent ratio by using a 10 μL injection volume. The sample was run for 30 min and analyzed with a 2489 UV/vis detector at 210 nm wavelength. Analyte identity was verified by using mass spectrometry. The resulting chromatogram was analyzed for the concentrations of betulin, betulinic acid, and lupeol.

#### 2.3.3 Scanning Electron Microscopy (SEM)

SEM samples were prepared by crushing dried extract samples and mounting them on sample stubs by using carbon tape. Samples were imaged using a Zeiss EVO 25 SEM instrument with a secondary electron detector.

### 2.4 Synthesis of Polyester Thermosets

Polyester thermosets were synthesized via melt polycondensation of pure or extracted betulin with DDDAc using glycerol as the cross-linker. For polymers synthesized using betulin-rich extracts, the amount of extract used was calculated to maintain an equimolar end group ratio based on the mass percent of hydroxyl groups for each extract (Table S1) such that each extract contributed 5.2 mmol of hydroxyl groups. For all samples, DDDAc (12.2 mmol, 2.76 g, 24.4 mmol –COOH groups) was fully melted in a 20 mL scintillation vial while stirring. Then, pure betulin (2.6 mmol, 1.13 g, 5.2 mmol –OH groups) or betulin extract (5.2 mmol –OH group) was added to the vial and allowed to fully dissolve while stirring. Glycerol (6.4 mmol, 0.59 g, 19.2 –OH groups) and dibutyltin dilaurate (∼0.4 mol %, 47 μL) were slowly added and the solution was stirred until homogeneous and reacted at 150 °C until viscous as noted by a qualitatively observed reduction in stir bar rotation speed. The viscous mixture was then poured into preheated (150 °C) PTFE molds (44.5 mm × 8 mm × 1.7 mm) that were pretreated with the PTFE release agent and promptly degassed in a vacuum oven for 20 min at 190 °C. Each sample was cured for 16 h at 150 °C. After curing, the samples were removed from the oven, allowed to cool to laboratory temperature, and cut in half prior to thermomechanical testing. Each sample was prepared in duplicate or triplicate. Polymer samples produced from extracts are referred to by the solvent/antisolvent extraction method used for its synthesis followed by “-P” to denote the polymer sample rather than the extract. Polymer samples produced from pure betulin sourced commercially are referred to as “Pure Betulin” in tables and figures throughout the manuscript.

### 2.5 Polyester Characterization

#### 2.5.1 Differential Scanning Calorimetry (DSC)

DSC measurements were performed using a TA Instruments Discovery Series DSC 2500. Samples of each polymer synthesized (5–10 mg) were sealed in Tzero aluminum pans, which were then punctured with a pinhole in the top to release water vapor. Samples were equilibrated at −50 °C under a N_2_ flow of 50 mL/min and heated to 200 °C at a rate of 10 °C/min before being cooled to −50 °C at the same rate. The obtained thermograms were analyzed for evidence of incomplete curing at and above the cure temperature (150 °C).

#### 2.5.2 Dynamic Mechanical Analysis (DMA)

A TA Instruments Q800 DMA instrument equipped with a gas cooling accessory was used to evaluate the thermomechanical properties of the synthesized polyesters. Samples were tested by using 0.10% strain amplitude at 1 Hz in a film tension clamp. Two heat–cool–heat temperature sweeps were completed at a heating rate of 3 °C/min and a cooling rate of 10 °C/min. Thermograms from the second temperature sweep were lightly smoothed using Universal Analysis software (TA Instruments) and analyzed. The glass transition temperature (*T*_g_) is reported as the temperature at which the tan δ trace reached a maximum on the second heating ramp. The cross-link density (*v*_e_) was calculated from the rubbery storage modulus (*E*′) according to eq 1:

1 $$v_{e}=\frac{E^{\prime }}{3RT}$$

where *E*′ is the storage modulus in the rubbery plateau, *R* is the ideal gas constant, and *T* is the absolute temperature corresponding to *E*′.^24^ Reported values contain averaged data from two to three independently prepared samples.

#### 2.5.3 Thermogravimetric Analysis (TGA)

The thermal degradation of the samples was measured using a TA Instruments Discovery Series TGA Q500. Samples of each polymer synthesized (5–10 mg) were placed in platinum sample pans containing disposal aluminum plans and heated from approximately 25 to 600 °C at a rate of 10 °C/min. Testing was completed under inert conditions using N_2_ and oxidative conditions using air. The sample gas flow rate was 60 mL/min, and the balance gas flow rate was 40 mL/min. The temperatures corresponding to 5% weight loss (*T*_5%_), which is reported as the initial decomposition temperature (IDT), and 50% weight loss (*T*_50%_) of the samples are averaged from two or three independently prepared samples.

## 3 Results and Discussion

### 3.1 Extraction Solvent Screening

Solvent selection is a multicriterion analysis that requires the evaluation of solvent function with regards to extraction efficiency/yield and selectivity as well as “green” attributes (environment, health, safety, life cycle impacts, etc.).^23,25,26^ Solvents for initial extraction screening were selected to include a range of readily available solvents with low hazard ranking as well as those from the literature that were shown to be effective for betulin extraction and/or had promising Hansen solubility parameters (Table S2).^27^ Chloroform was also evaluated as a comparison solvent since, despite its high hazard ranking, it is commonly used for extractions and has been shown to have a high yield for betulin from birch bark.^16^ Ethyl acetate/methanol mixtures were also evaluated as they have been shown to be effective at extraction of low-polarity compounds;^28^ a 2:1 (vol) ethyl acetate:methanol ratio was found to be most effective for sample prep and extraction.

The initial solvent screening evaluated which solvents had the greatest triterpenoid extraction yield (Figure S2). Extraction yield is defined here as the amount of betulin per mass of birch bark used, which is a relative value among solvents as it may vary depending on the birch bark source. The solvents with the greatest yields in a single extraction were 2:1 ethyl acetate:methanol, pure methanol, and chloroform. There was no apparent trend with Hanson solubility parameters, and the use of some solvents with disparate parameters resulted in high yields. For example, alcohols have high δ_h_ values compared to betulin yet were quite effective at extracting betulin. While 2:1 ethyl acetate:methanol is a relatively green solvent mixture, methanol is not typically renewably sourced, though routes for large-scale biomethanol production are currently being explored.^19^ Ethanol and ethyl acetate mixtures were tested instead in order to realize a fully biobased solvent mix.^22,29^ Combinations of ethanol and ethyl acetate were screened to obtain extraction yields comparable to that of 2:1 ethyl acetate:methanol, resulting in the adoption of 1:1 ethanol:ethyl acetate (EtOH:EtAC) as the mixture used for further process development.

### 3.2 Screening Supercritical Carbon Dioxide (scCO_2_)

Due to the promise for enhanced extraction, scCO_2_ was also evaluated for betulin extraction of birch bark.^30^ While pure scCO_2_ was ineffective at extracting betulin from birch bark, the use of modifiers (ethanol, ethyl acetate, and anisole) was evaluated to enhance the extraction yield. Anisole was found to be the most effective cosolvent modifier, but extraction yields using supercritical fluid extraction compared to multiple rounds of pure solvent extraction provided comparable yields (Figure S3). As such, the benefits of using scCO_2_ for extraction were deemed insufficient to warrant the use of this high-pressure technique.^17^

### 3.3 Extraction Solvent Process Development

Ethanol and EtOH:EtAc were evaluated for scale-up from the initial screening experiments using reflux boiling, a well-known, easily scaled, and effective extraction technique that was successfully applied for betulin extraction using ethanol.^17^ When applied to silver birch bark samples, this technique offered a relatively high betulin content in the solid (Table 1, samples EtOH and EtOH:EtAc). Previous literature has indicated that the use of solvents with intermediate polarity generally increases the content of triterpenoids in extracts; however, the selectivity was still relatively low as the betulin concentration of the extracts typically did not exceed 55–75%.^6^ In this work, the use of water as an antisolvent was adopted in order to facilitate product recovery as well as to increase triterpenoid content of the final solid product.

**Table 1:** Extract Triterpenoid Content

sample name	extraction solvent	solvent/antisolvent (water) ratio	betulin (wt %)	betulinic acid (wt %)	lupeol (wt %)
EtOH	ethanol	N/A	62.2 ± 1.9	1.7 ± 0.3	4.7 ± 0.3
EtOH/a	ethanol	1:1	74.9 ± 2.9	2.2 ± 0.1	6.0 ± 0.3
EtOH/EtAc	1:1 ethanol/ethyl acetate	N/A	69.7 ± 3.6	1.9 ± 0.2	4.6 ± 0.1
EtOH/EtAc/a	1:1 ethanol/ethyl acetate	1.5:1	81.5 ± 1.7	2.3 ± 0.1	4.1 ± 0.1

The use of water as an antisolvent caused a betulin-rich solid to precipitate from solution, allowing for the easy recovery of the solid and also an increase in the betulin content of the resultant extract (Table 1, samples EtOH/a and EtOH:EtAc/a). The addition of a polar antisolvent decreases the solubility of betulin (and other low-polarity components) in the liquid solvent and causes the precipitation of a betulin-rich solid. Because the solution is highly polar, polar impurities are less likely to be retained in the solid, thus resulting in an increase in purity. However, when adding water as the polar constituent, the phase behavior of the mixed solvent system must also be considered; while ethanol and water are miscible, ethyl acetate and water are only partially soluble. The solvent mixture must remain in a single phase to prevent the simple partitioning of betulin between the hydrophobic and hydrophilic layers of the two-phase system. Thus, various water:solvent ratios were tested to ensure that the solvent system (e.g., water:EtOH:EtAc) remained within the single-phase region of the ternary phase diagram.^31^

Model systems comprising solvent, water, and betulin were evaluated to determine which ratios resulted in maximal betulin recovery (Table S3). For ethanol, the 2:1 (volume solvent:volume water) ratio had the lowest recovery of betulin, while 1:1 had results similar to 1:2. In order to minimize water consumption, the 1:1 ratio was chosen for further process development. The effect of salt addition to the mixture was also tested to determine its impact on precipitation. There was no noticeable impact for both monovalent and divalent candidates (NaCl, CaCl_2_, KCl). Unfortunately, applying the model parameters to birch bark extraction gave rise to a trade-off between the yield and selectivity: the yield of betulin in the ethanol:ethyl acetate condition is lower, while the selectivity is highest. Yield may improve by incubating the solvent/water mixtures at depressed temperatures (1 h) before filtering out the precipitate (Figure 1) though the energy costs of cooling must be considered in the final process design. This incubation during the precipitation steps allows for improved extraction solid recovery at mildly depressed temperatures (6 °C).

**Figure 1:**
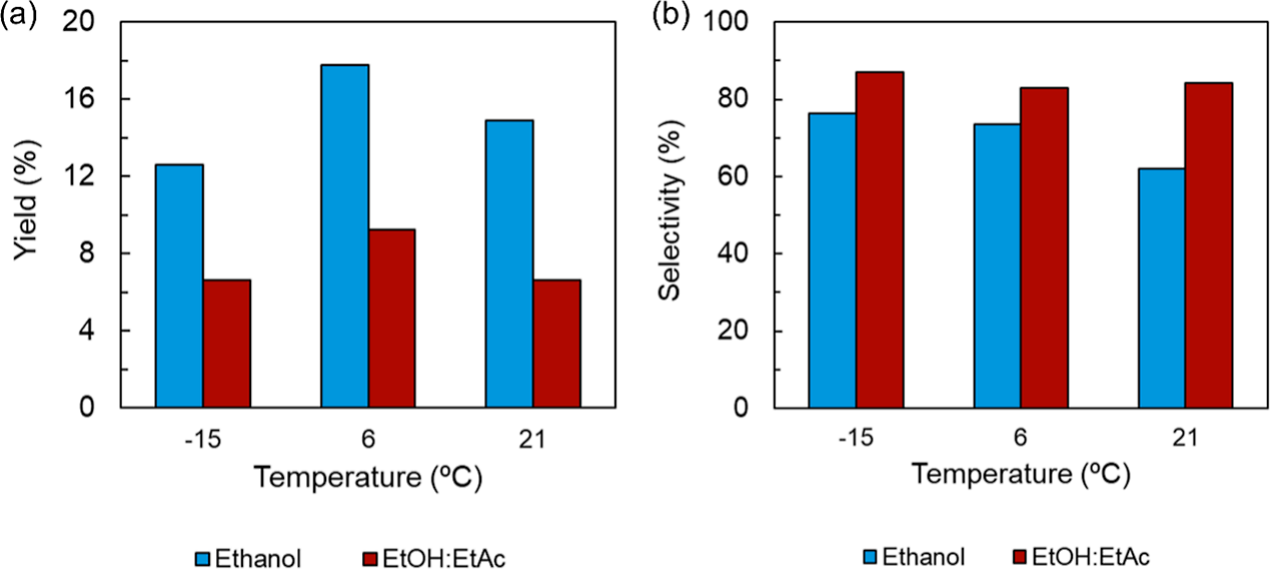
(a) Yield and (b) selectivity of betulin from extracts undergoing antisolvent precipitation followed by incubation of solvent/water mixtures (1 h) at the indicated temperatures. All extracts were first extracted via reflux boiling with either ethanol or ethanol:ethyl acetate.

The use of the 1:1 EtOH:EtAc solvent mixture resulted in extracts with significantly higher betulin content when compared to the use of only ethanol. As such, the sample yielding the highest betulin purity was made using EtOH:EtAc with antisolvent precipitation (EtOH:EtAc/a) at a 1:1 solvent:water volume ratio. While the selectivity of the ethanol extracts with and without precipitation aligns with the work of Fridén et al.,^17^ the added benefit of using the solvent mixture is that it provides extracted betulin selectivity >80% while maintaining the use of green solvents. Grazhdannikov et al.^18^ were able to obtain a higher selectivity (95–97%) using ethanol and ethyl acetate, separately, as solvents, but required a multistep process including saponification and purification with monoterpenes.

Other triterpenes, betulinic acid and lupeol, were also analyzed in the extract samples as they are known to be present at relatively high concentrations in birch bark. The ratio of betulin and betulinic acid is comparable for all samples (Table 1). While there is a small fraction of the overall extract, the proportion of lupeol is slightly higher in ethanol extracts compared to the EtOH:EtAc samples. The unknown fraction of the extracts potentially contains a variety of compounds, including triterpenoids, such as betulonic aldehyde and lupenone, as well as nontriterpenoids like suberin, phenolics, and various carbohydrates.^32^ These components could influence the polymerization process and the resulting polymer properties. Furthermore, suberic acid has the ability to cocrystallize with betulin in its pure form.^33^ Notably, suberic acid exhibits lower solubility in ethyl acetate compared to ethanol, which may have implications for its processing and application.^33^

SEM images show that the structures of the solid extracts may vary depending on the solid recovery method and extraction solvent (Figure 2). Samples obtained by simple solvent evaporation produced solids that were finer and were more fibrous in structure. The structure of samples obtained through antisolvent precipitation generally had larger masses. The EtOH/a sample had a flakey appearance whereas the EtOH:EtOH/a sample produced more sphere-like structures. It should be noted that for the antisolvent samples, unprecipitated betulin was not able to be recovered resulting in lower betulin yields. Bag and Dash^34^ found the self-assembly of betulin in various solvents—some solvents exhibited gel formation and created flower-like structures due to betulin self-assembly. The authors had also explored ethanol–water (1:1) mixtures but found that betulin remained a colloidal suspension (without self-assembly), indicating a lack of nucleation. Niu et al.^35^ also found evidence of betulin self-assembly into “hedgehog” morphologies induced through sonication to first create metastable aggregates and self-assembly via hydrogen bonding. In these studies, the precipitation conditions (solvent, use of sonication) were selected to induce a particular morphology in the resultant solids. For the polymers synthesized in this work, the superstructure is likely irrelevant, since the betulin was melted and dissolved before polymer synthesis.

**Figure 2:**
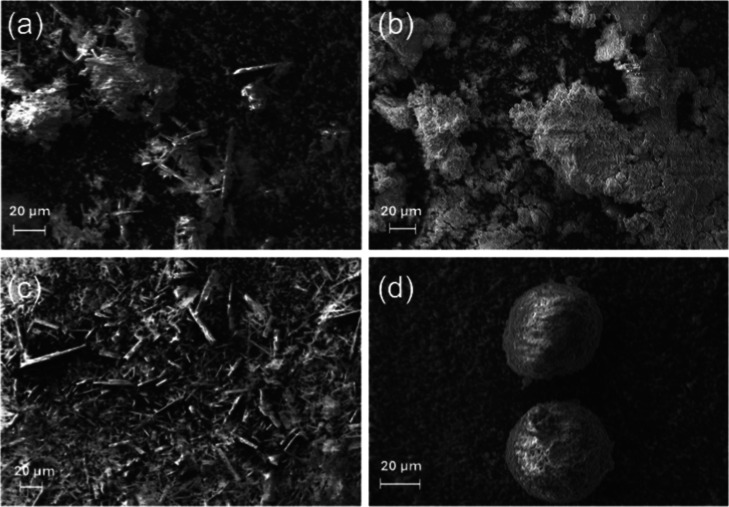
SEM images of dried extracts using (a) EtOH, (b) EtOH/a, (c) EtOH:EtAC, and (d) EtOH:EtAC/a. Scale bar is 20 μm.

### 3.4 Polyester Synthesis and Characterization

The four extracts were used to evaluate the feasibility of creating polyester thermosets directly from betulin-rich extracts, assess the properties of the resultant polymers, and compare them to polyester thermosets prepared from pure betulin sourced commercially. Specifically, the betulin-rich bark extracts were incorporated into polyester thermosets with biobased comonomers via melt polycondensation. As shown in Scheme 1, betulin-based polyester thermosets were synthesized with DDDAc as the comonomer and glycerol as the cross-linker in accordance with our prior method.^13,14^ When preparing thermosets using betulin-rich extracts or pure betulin, we used commercially sourced comonomers to isolate the effect of the betulin extraction process. Notably, this approach avoids the use of corrosive chemicals (e.g., acid dichlorides) and byproducts (e.g., HCl) and is carried out at a moderate temperature (∼150 °C) without the use of solvent. DSC experiments revealed no significant exotherm at and above the cure temperature, indicating that all samples were fully cured (Figure S4).

**Scheme 1:**
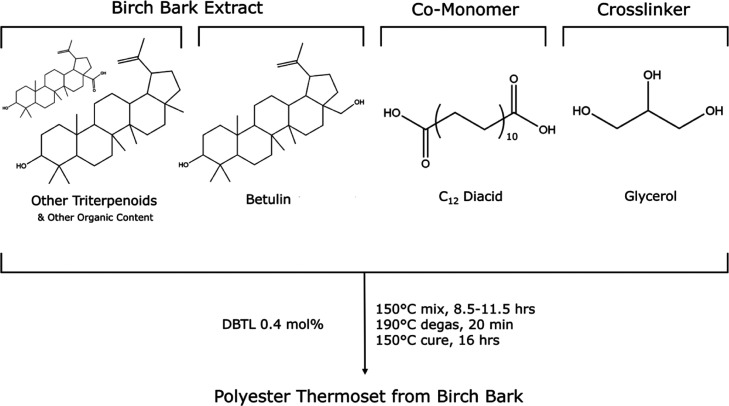
Melt Polycondensation for the Preparation of Thermosets from Birch Bark Extracts Rich in Betulin

Thermomechanical properties of the thermosets prepared using each extract were measured using DMA and compared to polyesters synthesized from pure betulin.^14^ The storage modulus (*E*′), loss modulus (*E*″), and tan δ curves are shown in Figure 3 and summarized results are available in Table 2. The average glass transition temperature (*T*_g_) ranges from 17 to 23 °C for samples synthesized from extracts compared to 22 °C for the sample prepared from pure betulin, indicating that all extracts yielded thermosets with similar *T*_g_s to thermosets made from pure betulin. The use of an antisolvent during extraction resulted in polymer samples (EtOH/a-P and EtOH:EtAc/a-P) exhibiting tan δ curves with lower peak heights compared to samples synthesized from extracts produced without the use of an antisolvent (EtOH-P and EtOH/EtAc-P). As the intensity of the tan δ peak reflects the extent of chain mobility throughout the glass transition,^36,37^ the lower tan δ peak exhibited by EtOH/a-P and EtOH:EtAc/a-P indicates less viscous behavior throughout the glass transition.^37^ In contrast, EtOH-P and EtOH/EtAc-P exhibit more dampening at their *T*_g_s. It is possible that the antisolvent refining step also concentrates components such as suberic acid^33^ and its shorter chain length results in decreased energy dissipation in these thermosets as observed previously.^13^ Further, higher concentrations of suberic acid in extracts purified with EtOH may also explain the lower tan δ peak in EtOH-P compared to EtOH/EtAc-P as suberic acid exhibits lower solubility in ethyl acetate compared to ethanol.^33^ Moreover, greater full widths at half-maximum (fwhm) of the tan δ curves corresponding to EtOH:EtAc/a-P indicate more heterogeneity in these polymer networks^37–39^ compared to EtOH:EtAc-P, despite the higher betulin content of the extracts purified using an antisolvent. This heterogeneity is consistent with higher concentrations of suberic acid in EtOH:EtAc/a-P due to the antisolvent refining step. However, no difference between the fwhm values for EtOH-P and EtOH/a-P was measured. Interestingly, a higher *E*′ in the glassy region is measured for EtOH-P compared to all other samples, perhaps owing to high-polarity compounds in these extracts that promote strong hydrogen bonding in the polymerized samples.^40^ The presence of these higher polarity compounds may also explain the greater fwhm of the tan δ curve corresponding to EtOH-P compared to EtOH/EtAc-P. We hypothesize that while refinement using an antisolvent following initial purification with ethanol in EtOH/a-P may remove these polar impurities, it may also concentrate suberic acid therefore resulting in no difference between the fwhm between the EtOH-P and EtOH/a-P. Notably, for samples synthesized from all extracts tested in this study, a rubbery plateau rather than a molten region was observed (Figure 3a), indicating the formation of cross-linked networks.^14,41^ We estimate cross-link density from the rubbery plateau storage modulus^36^ for each sample (Table 2). Qualitatively, the DMA thermograms corresponding to EtOH/EtAc-P are the most similar to those corresponding to networks from pure betulin and resulted in the highest estimated cross-link density. Overall, the polyesters synthesized from all birch bark extracts resulted in cross-linked samples with similar *T*_g_s to those synthesized with pure betulin. However, we did not observe that the use of an antisolvent to further purify the extract resulted in networks with properties closer to those synthesized from pure betulin, despite their higher betulin content. The antisolvent refining step may also concentrate components such as suberic acid^33^ that affect network properties, suggesting that the additional processing is not necessary.

**Figure 3:**
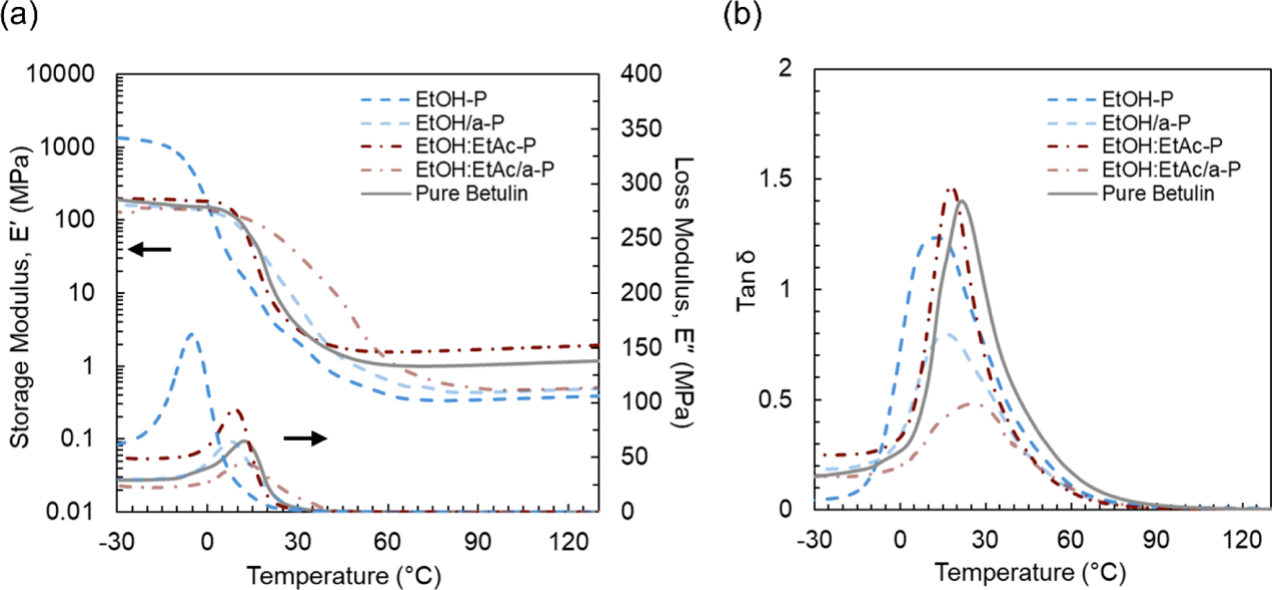
DMA of thermosets synthesized from birch bark extracts and pure betulin. (a) Dynamic moduli and (b) tan δ for thermosets prepared from each extract are shown and compared to samples prepared from pure betulin.

**Table 2:** Thermomechanical Properties of Thermosets Synthesized from Birch Bark Extracts and Pure Betulin^d^

thermoset samples	*E*′ at 25 °C (MPa)	*T*_g,tan δ_ (°C)^a^	tan δ peak height	fwhm (°C)^b^	rubbery *E*′ (MPa)^c^	ν_e_ (mol m^–3^)
EtOH-P	11 ± 7	17 ± 3	1.2 ± 0.2	37 ± 9	0.54 ± 0.4	61 ± 39
EtOH/a-P	19 ± 5	17 ± 1	0.75 ± 0.04	36 ± 2	0.38 ± 0.06	42 ± 7
EtOH/EtAc-P	4.7 ± 0.2	18 ± 1	1.5 ± 0.1	21 ± 1	1.4 ± 0.3	150 ± 32
EtOH/EtAc/a-P	43 ± 10	23 ± 2	0.48 ± 0.01	41 ± 1	0.61 ± 0.14	67 ± 15
pure betulin	7.0 ± 0.4	22 ± 1	1.4 ± 0.1	24 ± 1	1.0 ± 0.1	110 ± 10

a Temperature corresponding to the peaks in the tan δ thermograms.

b Full width half-maximum (fwhm) of the tan δ curve.

c Rubbery *E*′ at *T*_g,tan δ_ + 70 °C.

d Cross-link density at *T*_g,tan δ_ + 70 °C.

The thermal stability of these samples was measured using TGA in both an inert and an oxidative environment using N_2_ and air, respectively. Representative thermograms are displayed in Figure 4 and decomposition temperatures are summarized in Table 3. Samples prepared from pure betulin exhibited excellent thermal stability with initial decomposition temperatures (IDTs) occurring at 317 and 307 °C in inert and oxidative environments, respectively, while all thermosets prepared from extracts displayed IDTs ranging from 274 to 310 °C in N_2_ and 266 to 301 °C in air (Table 3). While these results indicate a trend toward slightly lower average degradation temperatures of samples prepared from the extracts compared to pure betulin, the differences are small such that the polymers prepared from extracts would likely be suitable in similar applications to those prepared from pure betulin. Qualitatively, the TGA thermogram corresponding to EtOH:EtAc-P is the most similar to those corresponding to networks from pure betulin, analogous to the DMA results. Our results suggest that the use of an antisolvent to further purify birch bark extracts does not enhance the thermal stability of the resulting thermosets and exhibits a trend toward lower IDTs despite the higher betulin content. Additionally, no samples show appreciable mass loss near 100 °C, indicating the water byproduct was removed from all samples during curing.

**Figure 4:**
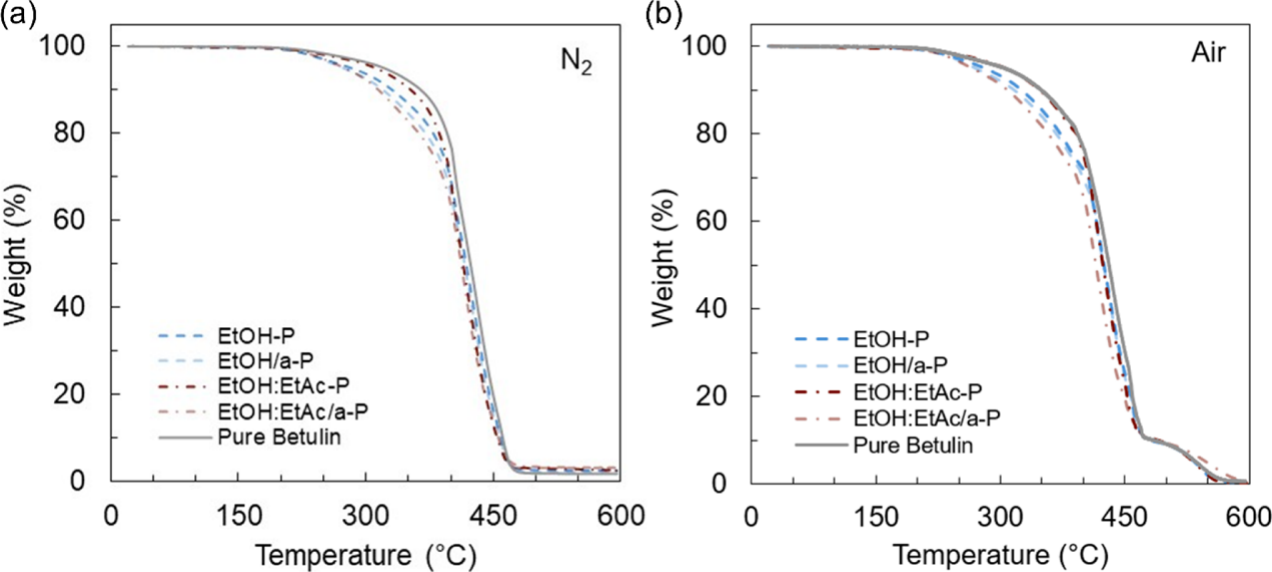
TGA thermograms of thermosets synthesized from birch bark extracts and pure betulin tested in (a) N_2_ and (b) air.

**Table 3:** Thermogravimetric Results for Thermosets Synthesized from Birch Bark Extracts and Pure Betulin

	N_2_			air		
thermoset sample	IDT (°C)	*T*_50_ (°C)	char yield^a^ (%)	IDT (°C)	*T*_50_ (°C)	char yield^a^ (%)
EtOH-P	285 ± 10	410. ± 5	2.83 ± 0.17	282 ± 10	418 ± 6	0.28 ± 0.10
EtOH/a-P	281 ± 7	417 ± 1	2.13 ± 0.03	275 ± 5	425 ± 1	0.43 ± 0.11
EtOH/EtAc-P	310 ± 2	415 ± 1	2.47 ± 0.08	301 ± 4	423 ± 1	0.17 ± 0.09
EtOH/EtAc/a-P	274 ± 1	412 ± 1	2.69 ± 0.51	266 ± 1	415 ± 1	0.45 ± 0.28
pure	317 ± 5	424 ± 1	3.70 ± 1.88	307 ± 3	431 ± 1	0.48 ± 0.05

a Char yield values determined at 595 °C.

## 4 Conclusions

In this work, we address a gap in the current literature connecting how the extraction process and extract composition affect the properties of biobased polymers and compare to polymers prepared from pure starting materials. Specifically, we developed a process to recover betulin-rich extracts from birch bark using green solvents, demonstrated the direct use of these extracts to create biobased polyester thermosets, and compared their properties to thermosets prepared from pure betulin. The process used ethanol and/or ethyl acetate as extraction solvents both of which can be derived from biomass. The applied water-based antisolvent procedure resulted in extracts with higher betulin contents (>80% selectivity). All birch bark extracts were used to prepare polyester thermosets via melt polycondensation with a biobased diacid and glycerol as the cross-linker, thus avoiding the use of solvents as well as corrosive chemicals and byproducts. Characterization of the resulting thermosets indicated that they were fully cured and that the water byproduct was removed from all samples during curing as evidenced through DSC and TGA experiments, respectively. Our results showed that the use of all four selected extraction procedures studied in this work yielded polyesters with *T*_g_s similar to that of samples made from pure betulin and are thermally stable up to 274 °C enabling their use in similar applications in which polyesters synthesized from pure betulin could be utilized. The results further indicate that the use of an antisolvent to further purify the extract may lead to more heterogeneity and less dampening in the resulting networks and that this further purification step does not significantly enhance their thermal stability despite the higher betulin content, suggesting that additional processing is not necessary. Additionally, avoiding the use of water as an antisolvent simplifies solvent recycling by eliminating the need to separate water and organic solvents. The holistic methodology we employ in this study may be used as a framework to probe how extraction method and solvent choice affect the properties of biobased polymers, thus addressing a current gap in the literature describing biobased plastics.

In our process, we have strived to adhere to the principles of green chemistry and engineering in the sourcing, extraction, and synthesis of biobased polymers (Tables S4 and S5). The extraction is simple, relying on a straightforward reflux boiling technique, an easily scalable technique, with green (and potentially bioderived) solvents without the need for extensive additional processing steps. The comonomers can be bioderived, and the solventless synthesis, which is carried out at moderate temperatures, avoids the use and formation of hazardous compounds. The polymerization is done via polycondensation and has a carbon economy of 100% with the resultant polyesters comprising 100% biomass-derived content (excluding the catalyst). The results of this study also highlight the potential of using mixed extracts directly as a polymer starting material, suggesting that highly refined betulin is not required for effective polymerization, which could significantly reduce the processing burden when starting with biomass feedstocks. By considering multiple life cycle stages, this study provides an example of how these principles can be incorporated into the development of biobased polyesters.

## Supplementary Material



## Notes

Additional experimental details, methods, and results, including a schematic overview of sample processing and extraction yields of betulin from atmospheric and scCO_2_-assisted extraction (PDF)
