# Supplementary material for: Thermosets from Birch Bark: A Holistic Approach Using Green Solvents and Processes
Source: ACS Omega. 2025 Jul 28;10(31):35207–16. doi: 10.1021/acsomega.5c05162 (PMC12355430; doi:10.1021/acsomega.5c05162)
Supplement: Supplementary file 1 [file ao5c05162_si_001.pdf]

Supporting Information

Thermosets from birch bark: A holistic approach using green solvents and processes

Megan L. Dodge[†], Luke Goodhope[†], Qwin Pisacane[§], Rongmin Tang[†], Sophia I. Harrill[†], Heather M. LaFrance[‡^], Alexandra M. Lehman-Chong[‡^], Joseph F. Stanzione, III [‡^], Lindsay Soh[†]*, Melissa B. Gordon[†]*

[†]Department of Chemical and Biomolecular Engineering, Lafayette College, 740 High Street, Easton, PA 18042, USA
[§] Integrative Engineering Program, Lafayette College, 740 High Street, Easton, PA 18042, USA
[‡]Department of Chemical Engineering, Rowan University, 201 Mullica Hill Road, Glassboro, New Jersey 08028, United States
^Advanced Materials & Manufacturing Institute (AMMI), Rowan University, 201 Mullica Hill Road, Glassboro New Jersey, 08028, USA

*Corresponding Authors: sohl@lafayette.edu; gordonm@lafayette.edu

This supplemental document contains 7 pages (Figures S1-S4 and Tables S1-S5).

Contents

1. Birch Bark Processing Overview
2. Extract Characterization
3. Green Solvent Parameters
4. Extraction yields of betulin from atmospheric and $scCO_2$ assisted extraction
5. DSC Thermograms
6. Alignment to Green Chemistry and Engineering Principles

1. Birch Bark Processing Overview

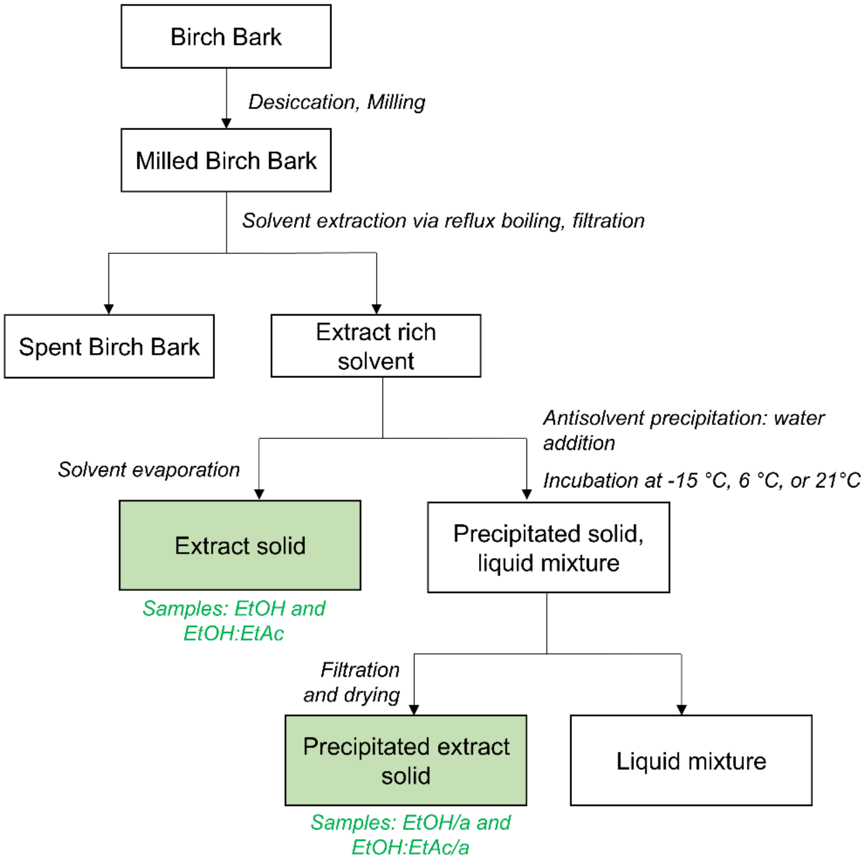

Figure S1: Schematic of sample processing for birch bark to extract samples

2. Extract Characterization

The hydroxyl number of each sample is calculated using the following equation

$$Hydroxyl\ number = \frac{(A - B) * N_t * 39.997}{W}$$

where
A = NaOH solution, mL, required for titration of the blank,
B = NaOH solution, mL, required for titration of the sample,
N_t = meq/mL of the solution at the temperature during analysis,
W = sample used, g, and,
39.997 = equivalent weight of NaOH.

The theoretical number of hydroxyl groups is calculated using the following equation

$$n_{hydroxyl} = 0.0098 * \left(\frac{M_n}{W}\right)$$

where
$n_{hydroxyl}$ = number of theoretical hydroxyl groups in the compound,
0.0098 = constraint that the sample size should be based on a maximum of 9.8 meq of hydroxyl groups present,
M_n = molecular weight of the hydroxyl-containing compound, and
W = sample used, g.

The hydroxyl-containing compound mass % is calculated using the following equation

$$Hydroxyl - containing\ compound, mass\ \% = \frac{(A - B) * N_t * M_n}{W * n_{hydroxyl} * 1000} * 100$$

where
A = NaOH solution, mL, required for titration of the blank,
B = NaOH solution, mL, required for titration of the sample,
N_t = meq/mL of the solution at the temperature during analysis,
M_n = molecular weight of the hydroxyl-containing compound,
W = sample used, g, and,
$n_{hydroxyl}$ = number of theoretical hydroxyl groups in the compound.

Table S1: Extract hydroxyl number and hydroxyl mass percentage

Sample Name	Extraction Solvent	Solvent: Antisolvent (water) Ratio	Hydroxyl Number	Hydroxyl Mass %
EtOH	Ethanol	N/A	73.9	9.5
EtOH/a	Ethanol	1:1	106.8	13.7
EtOH:EtAc	1:1 Ethanol: Ethyl Acetate	N/A	66.4	8.5
EtOH:EtAc/a	1:1 Ethanol: Ethyl Acetate	1.5:1	3.7	24.5

3. Green Solvent Parameters

Table S2: Green solvent class and Hansen solubility parameters for solvents used in this study

Solvent	Chemical Class	Rec*	δ_d	δ_p	δ_h
2-Methyl THF	Ethers	P	16.9	5	4.3
Acetone	Ketones	R	15.5	10.4	7
Acetonitrile	Aprotic polar	P	15.3	18	6.1
Anisole	Ethers	R	17.8	4.1	6.7
Chloroform	Halogenated	HH	17.8	3.1	5.7
Ethanol	Alcohols	R	15.8	8.8	19.4
Ethyl acetate	Esters	R	15.8	5.3	7.2
i-Propanol	Alcohols	R	15.8	6.1	16.4
Methanol	Alcohols	R/P	15.1	12.3	22.3
Methyl Acetate	Esters	P	15.5	7.2	7.6
Water		R	15.5	16	42.3
Betulin	Triterpenoid		17.69	3.75	9.67

*Rec: R = recommended, P = problematic, H = hazardous, HH = highly hazardous

4. Extraction yields of betulin from atmospheric and $scCO_2$ assisted extraction

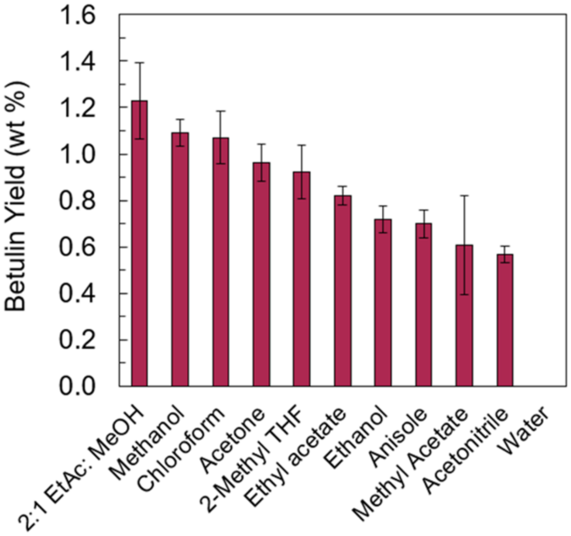

Figure S2: Betulin yield from atmospheric extraction using different solvents. Note that these screening experiments were conducted using river birch bark (*Betula nigra*) which is known to be notably lower in triterpenoid content compared silver birch; however, the obtained trends are expected to transfer among birch bark sources.

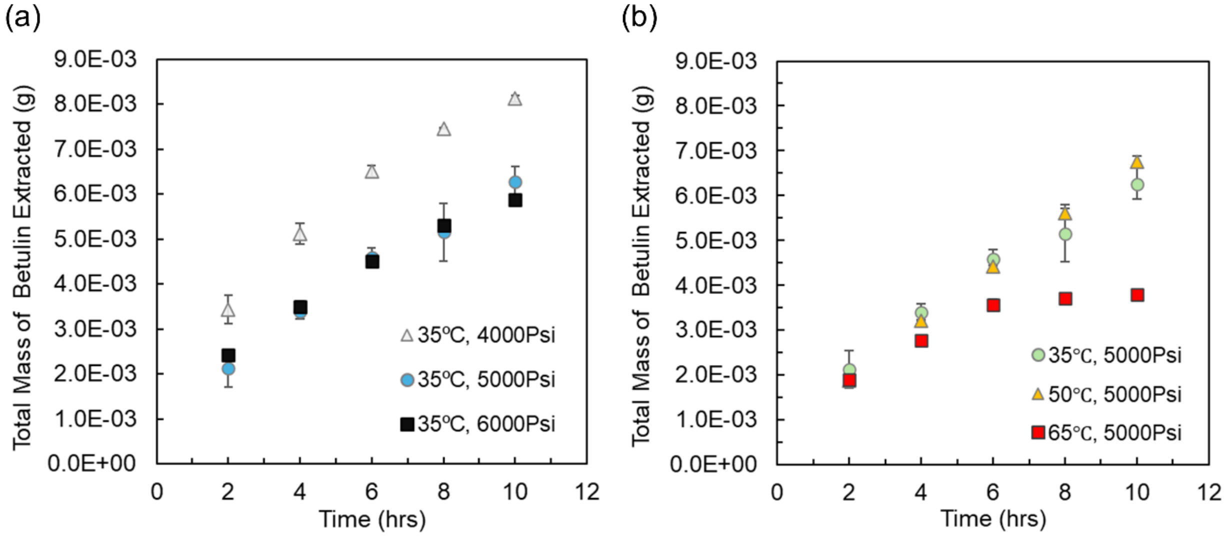

Figure S3: Extraction of betulin from 0.1 g of birch bark using $scCO_2$ assisted extraction with 8 vol% modifier (anisole) varying (a) pressure and (b) temperature. Extraction yield from unassisted extraction using pure modifier was 7.7 mg/0.1 g betulin.

Table S3: Antisolvent ratio recovery tests of pure betulin from solvent

Solvent	Solvent: Antisolvent (vol ratio)	Betulin Recovery (wt/wt)
Ethanol	1:1	0.978
Ethanol	2:1	0.833
Ethanol	1:2	1.000
1:1 Ethanol:Ethyl Acetate	2:1	0.517
1:1 Ethanol:Ethyl Acetate	1.5:1	0.704

5. DSC Thermograms

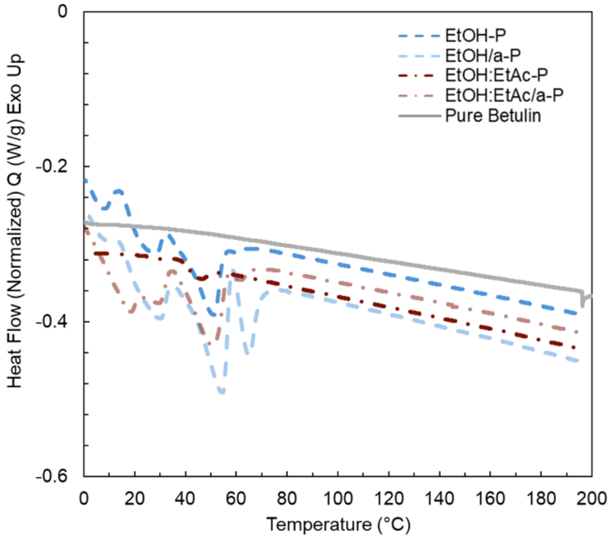

Figure S4: DSC thermograms of samples prepared from each extract and pure betulin

6. Alignment to Green Chemistry and Engineering Principles

Table S4: Alignment of this work to the Principles of Green Chemistry

Principles of Green Chemistry	Application to this work
Atom economy	All carbon atoms from the reaction feedstocks are incorporated into the final thermoset product.
Less Hazardous Chemical Synthesis	The polymerization reaction is solvent free and uses inherently benign starting materials.
Safer Solvents and Auxiliaries	The extraction utilizes greener solvents with low associated hazards.
Use of Renewable Feedstocks	All reaction starting materials can be sourced from biomass. The extraction solvents that were chosen can be biologically derived.
Catalysis	The use of a catalyst allows for relatively low reaction temperatures.
Inherently Safer Chemistry for Accident Prevention	Solvents were chosen for low hazard and high safety.

Table S5: Alignment of this work to the Principles of Green Engineering

Principles of Green Engineering	Application to this work
Designers need to strive to ensure that all material and energy inputs and outputs are as inherently non-hazardous as possible.	Solvents and starting materials were chosen for low hazard and high safety.
It is better to prevent waste than to treat or clean up waste after it is formed	The only reaction by-product is water.
Separation and purification operations should be designed to minimize energy consumption and materials use.	The overall process utilizes extracts directly from birch bark with minimal refinement.
Products, processes, and systems should be designed to maximize mass, energy, space, and time efficiency.	All carbon atoms from the reaction feedstocks are incorporated into the final thermoset product.
Design of products, processes, and systems must include integration and interconnectivity with available energy and materials flows.	The birch bark used as the process feedstock is often considered a waste product from the pulp and paper industry.
Material and energy inputs should be renewable rather than depleting.	All reaction starting materials can be sourced from biomass. The betulin-rich extract was sourced directly from birch bark. The extraction solvents that were chosen can be sourced from biomass.
